# The multifaceted roles of common gut microbiota in immune checkpoint inhibitor-mediated colitis: From mechanism to clinical application

**DOI:** 10.3389/fimmu.2022.988849

**Published:** 2022-09-14

**Authors:** Xu Han, Dan Zang, Dan Liu, Jun Chen

**Affiliations:** Translational Medical Research Center of Lung Cancer in Liaoning Province, Department of Oncology, The Second Hospital of Dalian Medical University, Dalian, China

**Keywords:** gut microbiota, immune-related adverse events, colitis, immune checkpoint inhibitor, immunity, mechanism, clinical application

## Abstract

With the arrival of the era of tumor immunotherapy, Immune Checkpoint Inhibitors have benefited countless tumor patients. However, the emergence of Immune-Related Adverse Events, especially Immune Checkpoint Inhibitor-Mediated Colitis (IMC), has become an important obstacle to immunotherapy. Therefore, it is very important to clarify the mechanism and influencing factors of IMC. The effect of gut microbiota on IMC is gradually becoming a research hotspot. Gut microbiota from different phyla can affect IMC by regulating innate and acquired immunity of tumor patients in various ways. In this review, we make a systematic and comprehensive introduction of the effect of gut microbiota on IMC. Through understanding the specific effects of gut microbiota on IMC, and then exploring the possibility of reducing IMC by regulating gut microbiota.

## Introduction

In recent years, the emergence of Immune Checkpoint Inhibitors (ICIs) has revolutionized the clinical treatment of malignant tumors. In particular, Cytotoxic T Lymphocyte Antigen-4 (CTLA-4) and Programmed Death-1/Programmed Death Ligand-1 (PD-1/PD-L1) have shown significant efficacy in the treatment of various tumors, thus becoming a new treatment method to prolong the survival of cancer patients (1). However, only a few patients obtained long-term clinical benefits, and many patients stopped treatment due to Immune-Related Adverse Events(irAEs), indicating irAEs are an important obstacle to immunotherapy (2). When ICIs launch immune attacks on normal tissues, irAEs will occur. These autoimmune toxicities can affect almost all organs. Different from other adverse drug reactions, irAEs have a broader time range of onset cycle, irAEs can occur as early as the first dose and a few months after treatment and even several months after the cessation of immunotherapy (3). Common irAEs include dermatitis, colitis, and thyroiditis, while others include pneumonia, hepatitis, nephritis, pituitary dysfunction, adrenal inflammation, and myositis. They also have terrible effects on the heart and central nervous system, and may even lead to death (2).CTLA-4 inhibitors and PD-1/PD-L1 inhibitors show different patterns of adverse events, indicating that they are different in maintaining immune tolerance (4). CTLA-4 inhibitors are prone to colitis, while PD-1/PD-L1 inhibitors have a high incidence rate of pneumonia and thyroiditis (5). Among irAEs, gastrointestinal irAEs are the most common cause of drug withdrawal in ICIs (6). Overall, the prevalence and severity of colitis caused by CTLA-4 inhibitors are higher than those caused by PD-1/PD-L1 inhibitors (7). However, with the more and more extensive use of combined immunotherapy, the frequency and severity of IMC have also greatly increased. For example, the incidence rate of IMC caused by CTLA-4 inhibitors and PD-1/PD-L1 inhibitors is 5.7%-39.1% and 0.7%-31.6% respectively, while the incidence rate of IMC caused by combination immunotherapy can reach 40.4% (8, 9). Although most irAEs, including IMC, can be alleviated to varying degrees by corticosteroid and biological agents, such as TNF-α antagonist. The dose and duration of drug use are positively correlated with the severity of IMC. But there are still many patients who are insensitive to corticosteroid and biological agents. Approximately 30%–60% of IMC patients appear recalcitrant to first-line glucocorticoid, showing no response to high-dose steroid within 3 days of onset or no complete response in 7 days (10). In addition, long-term corticosteroid exposure cause infection, hypertension, bone fracture, cataract, nausea, vomiting, and other gastrointestinal conditions, and metabolic issues and other side effects (11).To prevent side effects, the dosage and duration of corticosteroid and biological agents are strictly regulated and limited (12). Long-term and massive application of corticosteroid and biological agents also attenuates the antitumor effect of ICI (13, 14). Therefore, there is an urgent need for effective indicators of IMC and methods that can replace corticosteroid to improve patients’ compliance with immunotherapy.

Human gut microbiota is a complex system that affects the host’s nutrition acquisition, organ development, immune regulation, and other functions. With the development of analytical technology, people have further discovered the role of gut microbiota in maintaining the balance between human health and disease, particularly in promoting the development of the mucosal immune system and maintaining intestinal immune homeostasis (15–17). The composition of human gut microbiota remains relatively stable throughout life. *Bacteroidetes* and *Firmicutes* are the most abundant, accounting for about 90%, while *Proteobacteria*, *Actinobacteria*, *Fusobacteria*, and *Verrucomicrobia* are less abundant (18). The change of gut microbiota has a profound impact on the host, not only affecting the intestinal tract but also affecting many organs of the whole body (19). Generally speaking, the highly diverse gut microbiota establishes a symbiotic relationship with the host immune system to maintain the homeostasis of the host, and the breaking of this homeostasis can lead to chronic inflammation, autoimmune diseases, and even tumors. Our previous studies have revealed that gut microbiota may influence tumor progression, treatment and prognosis (20–22).

In recent years, cancer immunotherapy brought significant improvements for patients in terms of survival and quality of life. However, there are obvious individual differences in response to immunotherapy, and not all patients can obtain lasting benefits due to the occurrence of irAEs. Considering the close relationship between gut microbiota and the immune system, more and more studies have focused on the role of gut microbiota in regulating the efficacy of tumor immunotherapy and reducing side effects. Many studies have shown that the composition of gut microbiota can regulate the host response to ICIs, and higher microbial diversity is related to better treatment outcomes (23). The composition of gut microbiota is different between responders and non-responders to immunotherapy (24). Fecal microbiota transplantation (FMT) of responders showed improved response to ICIs (25). In conclusion, the efficacy of ICIs is related to the composition of gut microbiota, and specific bacteria can effectively improve the therapeutic response of ICIs.

Because of its complex effects on the human body, the gut microbiota has been regarded as a new “organ” of the human body and a new target for drug development. The gut microbiota is a very promising research field in reducing irAEs. Recently, several scientific studies on the impact of gut microbiota on IMC have revealed the relationship between the two, which may open up a new way to prevent and improve IMC. This review summarizes the related studies on the pathogenesis of IMC, especially the effect of gut microbiota. Furthermore, we also described the possible mechanism and clinical application of gut microbiota affecting IMC to explore the possibility of the gut microbiota as a biomarker and treatment of IMC.

## Immune checkpoint inhibitors

At present, the most widely used immune checkpoint inhibitors are PD-1, PD-L1and CTLA-4 inhibitors. PD-1 presents in T cells, B cells, tumor-infiltrating lymphocytes, myeloid cells. PD-L1 exists on the surface of both antigen-presenting cells (APCs) and tumor cells, resulting in weakening of immune surveillance after binding with PD-1. CTLA-4 is typically expressed on T cells, B-cell subsets, and thymocytes, which is associated with the suppression of T-cell activity. T-cell activation results in enhancing immune response and CTLA-4 and PD-1 expression. CTLA-4 is constitutively located on the surface of Foxp3 CD4 regulatory T (Treg) cells, harbors higher affinity than CD28, contributing to competitively bind CD80/86, thus attenuates T cell activation (26). In addition, PD-1 with PD-L1 suppresses the T-cell receptor (TCR) downstream signaling, enhanced the inhibitory effect of Treg cells (27–29). Both CTLA-4 and PD-1/PD-L1 inhibitors can strengthen the activation and proliferation of effector T cell, restrain Treg cell function, induce inflammatory cytokines, boost antitumor immunity.

More and more studies have focused on the interaction between gut microbiota and ICIs, and the positive role of gut microbiota in enhancing response to ICIs has also been proved. *Bacteroidales, Faecalibacterium* play key roles in the immunostimulatory effects of CTLA-4 blockade (30). Key bacterial species belonging to various phyla, including Actinobacteria (*Bifidobacteriaceae* spp. and *Coriobacteriaceae* spp.) and Firmicutes (*Ruminococcaceae* spp. and *Lachnospiraceae* spp.), *Verrucomicrobia* (*Akkermansia muciniphila*) are associated with favorable response to PD-1 inhibitors by increasing the recruitment of T cells in cancer patients (31, 32). *Bifidobacterium* enhanced the effect of PD-L1 inhibitor, augmented dendritic cell function leading to enhanced CD8(+) T cell priming and accumulation in the tumor microenvironment (33).

## Immune checkpoint inhibitor-mediated colitis

IMC is a common irAE and mortality rate of about 5%which sometimes terminates the treatment process of cancer patients (2). Meta-analysis of 629 patients from 26 studies showed that ICIs was permanently discontinued in 52.4% of patients with IMC (34). Colitis accounts for 37% of deaths caused by irAEs (35). Death cases due to irAEs treated with anti-CTLA-4 are usually from IMC, while death cases of irAEs related to anti- PD-1/PD-L1 are usually from pneumonia. irAEs with the most common and highest mortality after combined immunotherapy are also IMC (2). Because IMC has no specific biomarkers, the preliminary diagnosis can be made only when symptoms and imaging support are presented. IMC can appear at any time during the treatment process, so it is difficult to accurately predict the onset time of IMC (36). The onset time of IMC ranges from 0 to 27 weeks and may vary by ICIs type. Colitis caused by CTLA-4 inhibitors appears to occur later than that caused by PD-1/PD-L1 inhibitors, with a median onset time of about 6 to 7 weeks (8). The onset time of colitis caused by PD-1/PD-L1 inhibitors ranged from 0.6 to 119.9 weeks after treatment. In addition, the onset time of IMC was significantly earlier in patients treated with combined ICIs (37). Studies have observed that the regression time of IMC is more than 18.5 weeks, which is more than 10 times the half-life of CTLA-4 inhibitors (8). This means that the impact of immunotherapy on the human immune system is long-term, so the occurrence of related symptoms should be monitored for a long time after the use of ICIs. The clinical symptoms of IMC include diarrhea, abdominal pain, bloody stool, fever, nausea, vomiting, and loss of appetite, among which diarrhea is the most common symptom. Severe IMC can also lead to intestinal perforation, toxic megacolon, and even life-threatening (38). The common CT findings of IMC are mesenteric vascular congestion, intestinal wall thickening and fluid-filled colon dilatation (39). IMC endoscopy usually shows mucosal ulcer, and sometimes nonulcerative inflammatory morphology such as erythema, exudation, erosion, loss of vascular pattern, edema, or granular mucosa (40). The pathological manifestation of IMC is that lymphocyte infiltration leads to intestinal lamina propria expansion, intraepithelial neutrophilia, and neutrophil crypt abscess (41). Generally, IMC is diagnosed after excluding infection, inflammatory bowel disease(IBD) and tumor metastasis. Endoscopic biopsy is considered the gold standard for IMC diagnosis (8). IMC is usually treated with corticosteroid, but many patients are not sensitive to corticosteroid. At the same time, due to the obvious side effects of corticosteroid on the whole body, they cannot be used in large quantities for a long time. Therefore, it is urgent to develop new treatments. Although increasing experience with IMC therapy has improved the clinical diagnosis and monitoring of IMC, it remains one of the main obstacles in immunotherapy.

## Mechanism of IMC

**Figure 1:**
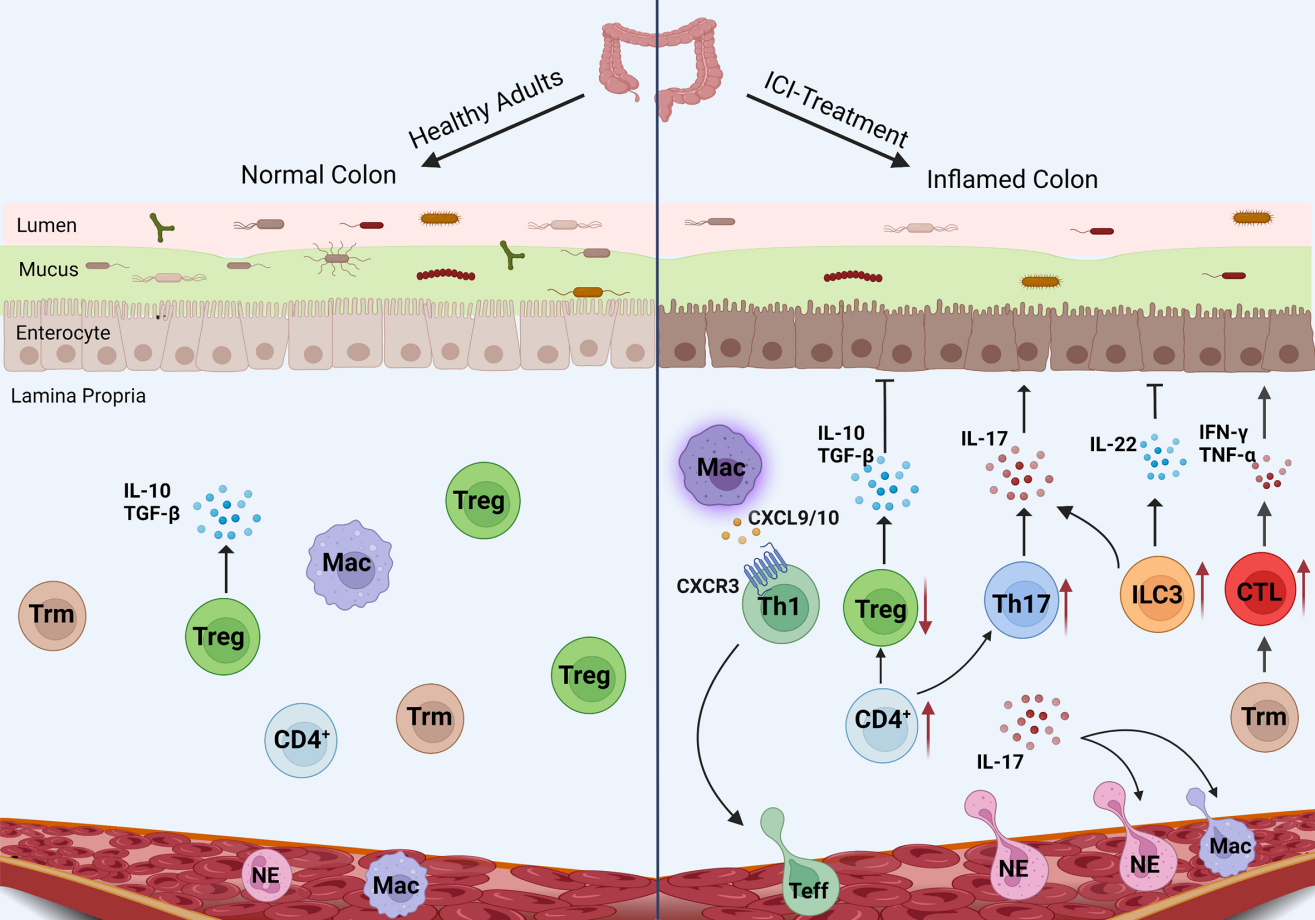
Mechanism of IMC. The pro-inflammatory pathways (CTL, Th17 cells, and neutrophils) enhance in IMC, while the anti-inflammatory pathways (Treg differentiation and IL-10 secretion) are inhibited. Macrophages recruit T cells through chemokines, and ILC3 plays an unknown role in IMC.

irAEs are the result of the destruction of self-tolerance and the pathogenesis of irAEs involves many factors. As an organ-specific irAE, IMC is mainly induced by the recruitment of T cells and the production of inflammatory factors. However, there are many mechanisms different from irAEs in other organs. An in-depth understanding of these pathogenic mechanisms will help us to comprehensively understand IMC. Innate and adaptive immune abnormalities as well as changes in gut microbiota can affect the occurrence and development of IMC (**Figure 1**).

### Innate immune abnormality

Neutrophils are the most abundant type of white blood cell in the human body. In the acute stage of inflammation, neutrophils are the first echelon to migrate to the inflammatory site. Pathological examination shows IMC has obvious neutrophil infiltration in the intestinal lamina propria. Analysis of the gene expression profile in whole blood samples from melanoma patients treated with ICIs showed that neutrophil activation markers CD177 and CEACAM1 were highly expressed in patients with gastrointestinal adverse reactions at baseline and continued to rise during the course of treatment (42). Group 3 innate immune cells (ILC3) are mainly concentrated in the intestinal tract, which produce immune responses to bacteria and regulate the balance of microbiota. The main homeostasis cytokine produced by ILC3 is interleukin-22(IL-22), which maintains intestinal homeostatic and promotes the proliferation of intestinal stem cells through IL-22 (43). On the contrary, ILC3 can also promote IL-17 production. The recent research has demonstrated that the severity of IMC is positively correlated with the number of ILC3 (44). Therefore, the current research shows that the role of ILC3 in IMC is contradictory and needs more detailed research to clarify. Another kind of innate immune cell is mucosa-associated invariant T(MAIT) cells. The number of MAIT cells increases in the intestinal tissues of patients with IMC, but there is no significant change in patients without IMC and patients with ulcerative colitis (45). T cells in IMC express high levels of IFN-γ、CXCR6 and CXCR3. CXCL16 is a ligand of CXCR6, which is expressed by myeloid cells in the colon and its expression is regulated by IFN-γ And TNF-α (46). Their binding also contributes to tumor cell metastasis, so they are potential targets to improve IMC (47). CXCR3 is a key surface molecule of Th1 cells, CXCL9 and CXCL10 as ligands of CXCR3 are induced by IFN, and its encoding genes are overexpressed in myeloid cells of IMC cases, especially macrophages, which help to recruit effector T cells to inflammatory sites (46). Studies have shown that CXCR3-deficient mice will not develop colitis induced by dextran sodium sulfate(DSS), confirming the influence of this pathway in the development of colitis (48).These studies suggest that myeloid cells, especially macrophages, play a significant role in T cell recruitment during the pathogenesis of IMC.

### Adaptive immune abnormality

High levels of infiltration of CD4+T cells and CD8+T cells occurred in IMC cases. CD4+T cells are the main T cell subset in CTLA-4-induced colitis patients, while CD8+T cells are dominant in PD-1-induced colitis. TNF-α is highly expressed in intestinal mucosa with CTLA-4-induced colitis (49, 50). Single-cell analysis of IMC patients also confirmed that the number of cytotoxic T lymphocytes (CTL) and circulating T cells increased, and tissue resident memory T cells (Trm) decreased, while the colon Trm cells of ICIs-treated patients without IMC did not change. TCR sequences of T cells overlapped between CD8+ Trm cells and CTL, indicating the differentiation of CD8+ Trm cells into CTL. At the same time, the content of granzyme B and IFN-γ produced by CD8+ Trm cells increased. It can be speculated that high levels of CD8+ Trm cells in the colon are involved in the pathogenesis of IMC, and its cell activation promotes the rapid recruitment of CD8+ T cells and enhances the damage to the colon mucosa (46, 51). This also explains that IMC is more acute than other types of colitis. α4β7 and αEβ7 integrins are expressed on intestinal mucosal intraepithelial lymphocytes (IEL) and play a crucial part in the homing and localization of lymphocytes to the intestinal mucosa (52). Genes Coding αE、α4 and β7 (ITGAE 、ITGA4、ITGB7) are highly expressed in IMC-related T cell populations, especially CD8+ T cells, indicating that this may be a potential target for the treatment of IMC. And clinical studies have demonstrated the use of α4β7 integrin inhibitor (vedolizumab) in the treatment of hormone-resistant IMC patients (53).

Regulatory T cells are T cell subsets that control autoimmunity *in vivo*, which can maintain immune tolerance and inhibit inflammation. Treg cells overexpressed CTLA-4, so CTLA-4 inhibitors can induce the inactivation of related intratumor and intestinal CTLA-4+treg cells, and induce the activation of effector T cells, resulting in the development of IMC (54). Chaput et al. observed that IMC patients had a low baseline level of sCD25. CD25 is the α chain of IL-2 receptor and CD25 is important for IL-2 in promoting the development of Treg cells (55). In the single-cell analysis of IMC patients, the number of Treg cells did not decrease, but the Treg cell’s gene expression profile changed. It is speculated that IMC may not be caused by the consumption of CTLA-4+Tregs, but Treg cells may show a Th1-like pro-inflammatory effect under the action of IFN- γ (46).

A clinical trial analyzing gene expression in peripheral blood showed that CCL3, CCR3, IL-5, and IL-8, which are mainly involved in inflammatory immune response, are closely associated with immunotherapy-related diarrhea, especially grade 2-4 diarrhea (56). The expression of IL-8 is regulated by IL-17. IL-17 is a pro-inflammatory cytokine, mainly produced by Th17 cells, which participates in the pathogenesis of various autoimmune diseases and promotes tumor progression. The baseline level of IL-17 is related to severe immunotherapy-related diarrhea. More importantly, serum IL-17 level has been elevated throughout the course of IMC (57, 58). The pathogenicity of Th17 cells is more prominent when the Treg cell’s function is limited. IL-10 is an important cytokine for inhibiting colitis which is secreted by Treg cells. When the IL-10 receptor (IL-10R) gene in Treg cells in the mouse model is knocked out, it will develop into colitis under the action of Th17 cells, highlighting the indispensable role of IL-10 in maintaining the stability of the intestinal environment (59). In addition, IL-17 stimulates intestinal epithelial cells (IEC) to express chemokines CXCL1, CXCL2, CXCL5, CXCL8 and granulocyte-macrophage colony-stimulating factor(GM-CSF), which attract neutrophils and prevent their apoptosis cause neutrophil infiltration (60–62). Intraepithelial neutrophilia granulocytosis is also a typical pathological manifestation of IMC. Furthermore, it acts systemically by increasing serum amounts of granulocyte-colony-stimulating factor (G-CSF) and GM-CSF to increase the numbers and speed the development of neutrophils and macrophages. IL-17 also can facilitate the recruitment of large numbers of immune cells through the induction of matrix metalloproteinases (63). In conclusion, the Th17/IL-17 axis affects IMC in many ways.

### Bidirectional regulation of IMC by gut microbiota

In addition to the pathogenesis discussed above, the gut microbiota is also actively involved in the pathogenesis of IMC. Understanding the impact of gut microbiota on human immunity is crucial for the treatment and reestablishment of protective gut microbiota to prevent and improve IMC. The human gut microbiota is known to alter immune homeostasis and tolerance. The diversity and composition of microorganisms may affect IMC by changing the immune components in tumors and circulation (**Table 1**).

**Table 1:** Microbiota associated with IMC.

Role	Bacterial phylum	Bacteria	Association with IMC outcomes	ICI treatment	Refs
Positive	Bacteroidetes	*Bacteroides fragilis*	Reduced colitis symptoms caused by CTLA-4 inhibitors	Anti-CTLA4 ab	(30)
	Bacteroidetes	*Bacteroidaceae, Rikenellaceae, Barnesiellaceae*	Higher in patients who did not develop IMC.	Anti-CTLA4 ab	(64)
	Bacteroidetes	Not specified	Higher in patients who did not develop IMC.	Anti-CTLA4 ab	(55)
	Bacteroidetes	*Bacteroides vulgatus*	Associated with lower incidence of irAEs	Any	(65)
	Firmicutes	*Lactobacillaceae*	Associated with low-grade irAEs	Anti-PD-1/L1 ab	(66)
	Firmicutes	*Negativicutes class, Veillonellaceae family*, *Dialister genus*	Enriched in patients with mild IMC	Anti-PD-1/L1 ab	(67)
	Proteobacteria	*Raoultella*	Associated with low-grade irAEs	Anti-PD-1/L1 ab	(66)
	Proteobacteria	*Desulfovibrio*	Significantly associated with lower incidence of irAEs	Any	(68)
	Proteobacteria	*Enterobacteriaceae*	Associated with clinical remission of IMC	Any	(69)
	Gracilicutes	*Burkholderia cepacia*	Reduced colitis symptoms caused by CTLA-4 inhibitors	Anti-CTLA4 ab	(30)
	Verrucomicrobia	*Akkermansia*	Related to low-level irAEs	Anti-PD-1/L1 ab	(66)
	Actinobacteria	*Bifidobacterium*	Associated with low-grade irAEs	Anti-CTLA4 ab	(70)
	Actinobacteria	*Bifidobacterium*	Significantly associated with lower incidence of irAEs	Any	(68)
Negative	Bacteroidetes	*Bacteroides intestinalis*	Enriched in patients with above grade 3 IMC	CICB	(71)
	Bacteroidetes	*Prevotellamassilia timonensis*	Enriched in patients with severe IMC	Anti-PD-1/L1 ab	(67)
	Bacteroidetes	*Bacteroides dorei*	Significantly enriched in patients prone to irAEs	Any	(65)
	Firmicutes	*Faecalibacterium prausnitzii*	Higher in patients who developed IMC.	Anti-CTLA4 ab	(55)
	Firmicutes	*Agathobacter*	Enriched in patients with severe irAEs	Anti-PD-1/L1 ab	(66)

It was found that CTLA-4 inhibitor treatment in mice could lead to intestinal injuries, such as increased IEC death, imbalance of IEC and IEL homeostasis, and even colitis, and this effect was mediated by gut microbiota. Mice treated with *B. fragilis* and *Burkholderia cepacia* reduced symptoms of colitis caused by CTLA-4 inhibitors and enhanced the therapeutic effect (30). Dubin et al. studied melanoma patients treated with anti-CTLA-4 and found the fecal samples of patients without IMC had higher bacterial richness and more *Bacteroidetes* phylum. Furthermore, a model based on bacterial function in feces was proposed to predict the risk of IMC in patients with melanoma (64). Chaput’s study also showed that in melanoma patients receiving anti-CTLA-4 treatment, different gut microbiota composition was related to the occurrence and development of IMC and clinical efficacy of ICIs. The bacterial diversity in the intestines of patients with IMC is reduced, and Firmicutes content is rich in patients prone to IMC, while the proportion of *Bacteroidetes* in patients without IMC is higher (55). Other studies have shown that the use of *Bifidobacterium* can reduce IMC without damaging the anti-tumor function of CTLA-4 inhibitors (70).

The baseline fecal microbiome composition of lung cancer patients undergoing irAEs including IMC was significantly different from other lung cancer patients. *Bifidobacterium* and *Desulfovibrio* are significantly associated with a lower incidence of irAEs, and this observation is consistent across all severity levels of irAEs. Dynamic longitudinal monitoring of the gut microbiome in lung cancer patients showed the microbiome α-diversity decreased could be observed at the onset of irAEs. With the cessation of ICIs and hormone treatment, the symptoms of irAEs gradually reduced or even disappeared, and the microbiome α-diversity also showed decline slows down or completely reversed to the baseline (68). Analysis of baseline gut microbiota in advanced non-small cell lung cancer patients who received PD-1/PD-L1 inhibitors showed that intestinal microbiota was related to irAEs including IMC, among which *Lactobacillaceae*, *Raoultella*, and *Akkermansia* were related to low-grade irAEs, while *Agathobacter* was enriched in the feces of patients who experienced more severe irAEs (66). A stool analysis of patients with hepatobiliary carcinoma who developed IMC after receiving a PD-1/PD-L1 inhibitor showed that the *Negativities* class, *Villonellaceae* family, and *Dialist* genus of *Firmicutes* phylum were enriched in patients with mild IMC, while the dominant species in the feces of patients with severe IMC was *Prevotellamassilia timonensis* from the *Bacteroidetes* phylum (67). Interestingly, this was contrary to the previous research results. Although many studies have confirmed that the existence of *Bacteroidetes* can reduce the occurrence of irAEs, it seems that different species of *Bacteroidetes* play different roles in irAEs. *Bacteroides dorei* is significantly enriched in melanoma patients prone to irAEs, and *Bacteroides vulgatus* is associated with a lower incidence of irAEs (65). In addition, studies have found that *Enterbacteriaceae* is associated with clinical remission of IMC, and medium-grade IMC often indicates a better anti-tumor effect (69).

Combined immune checkpoint blocking (CICB) therapy targeting CTLA-4 and PD-1 is associated with high clinical benefits across tumor types, but unfortunately, the incidence of irAEs is also higher with the widespread use of CICB. Baseline *Bacteroides intestinalis* abundance was higher in gut microbiota samples from patients with IMC above grade 3 than below grade 3. The mouse model also showed similar results. The administration of CICB increased the content of *Bacteroides intestinalis*, and the mice with *Bacteroides intestinalis* suffered serious ileum damage (71).

### Nutrients, genetic susceptibility and other factors

Although immune system abnormality is the main cause of IMC, the pathogenesis of IMC is complex and diverse, and also involves nutrients, genetic susceptibility and other factors.

A retrospective study found that C-reactive protein (CRP) was elevated in patients with irAEs including IMC, but the elevated level of CRP had nothing to do with the severity of inflammation. Therefore, it was inferred that the elevated CRP could predict the occurrence of irAEs in the absence of infectious diseases (72). Elevated CRP baseline level is positively correlated with the infiltration of CD8+ T cells. It is known that activated effector T cells can activate a systemic inflammatory response, which may be the link between CRP and irAEs (73). The use of vitamin D before ICIs treatment significantly reduced the incidence of IMC, and the immune cells of vitamin D deficient mice showed IL-17 and IFN-γ increased secretion and destruction of epithelial barriers leading to IMC (74, 75). A higher baseline level of soluble CTLA-4 (sCTLA-4) is closely associated with irAEs in patients with melanoma, especially in the gastrointestinal tract. sCTLA-4 is mainly produced by Treg cells and participates in immune escape, but it is inconsistent with the above results. In conclusion, the feasibility of SCTLA-4 as a biomarker for predicting IMC needs further exploration (76). Genetic susceptibility is a key factor in autoimmune susceptibility. Human Leukocyte Antigen (HLA) haplotypes and polymorphisms of immunomodulatory genes such as CTLA-4 and CTLA-1 are one of the leading causes of various autoimmune diseases and may participate in the occurrence and development of irAEs. Through the analysis of HLA allele typing, it is determined that there is a significant correlation between HLA-DQB1*03:01 and IMC[50]. There may be a common antigen between tumor and colon tissue, which enables colon tissue to be recognized by antigen-specific T cells, resulting in colon injury (64, 77).

Innate and acquired immune complement each other in the human body and jointly maintain immune homeostasis. The application of ICIs will interfere with the balance between inflammation and tolerance, leading to abnormal immune system. Resulting in enhanced pro-inflammatory pathway and inhibited anti-inflammatory pathway in the intestine, which is manifested in active T cell recruitment mediated by myeloid cells, increased differentiation of pro-inflammatory cells and release of pro-inflammatory factors, and decreased differentiation of anti-inflammatory cells. These immune system disorders can induce the development of IMC. In addition, nutrients and genetic susceptibility are also the key factors of IMC.

## Possible mechanism of gut microbiota and metabolites affecting IMC

**Figure 2:**
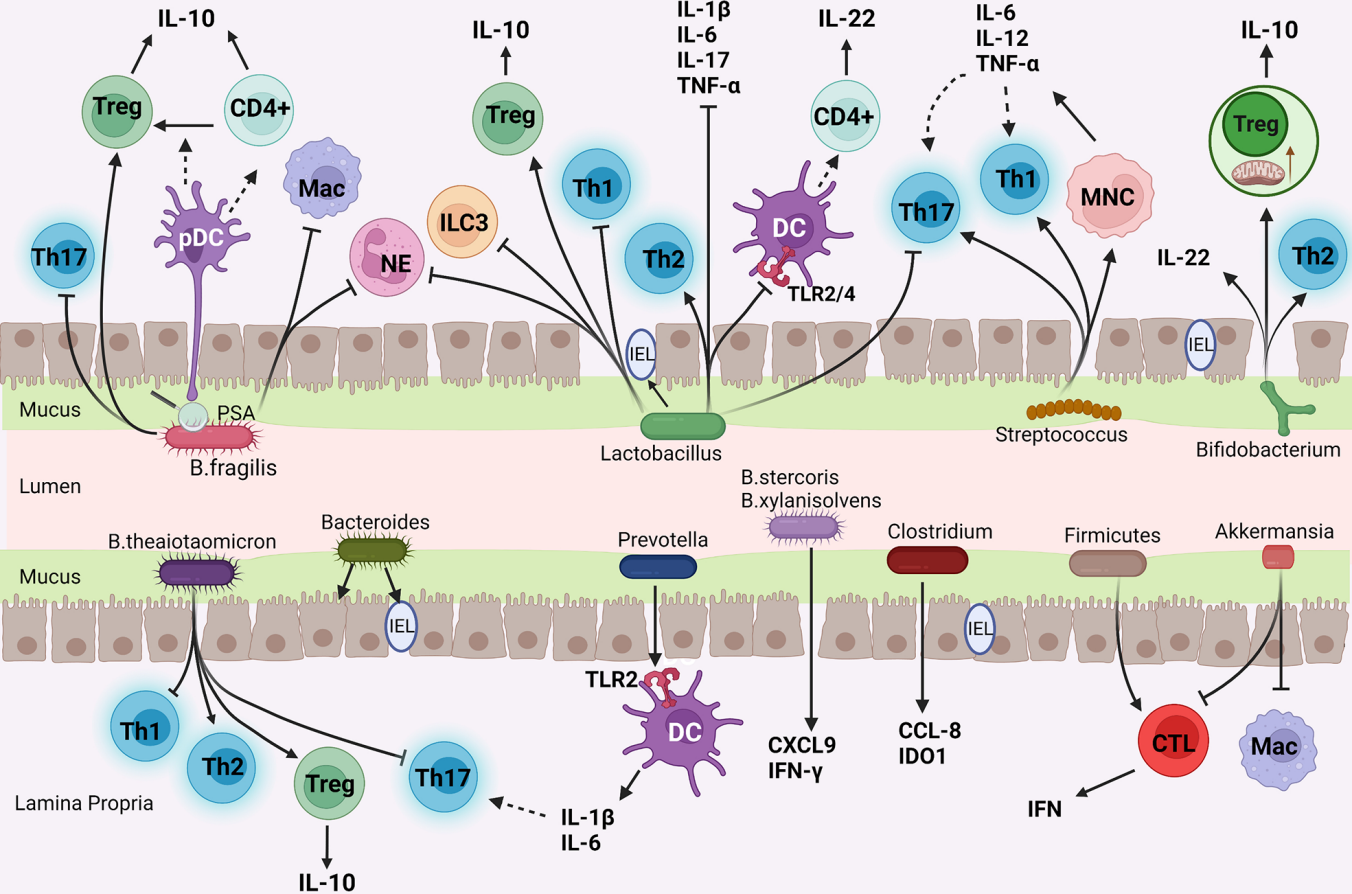
The mechanism of common gut bacteria in colon affecting IMC. (I) *Bifidobacterium* enhances Treg cells function and mitochondrial activity; (II) *Bacteroides* and *Lactobacillus* inhibit pro-inflammatory cells (Th1, Th17 cells, and neutrophils) and enhance anti-inflammatory pathways (Treg, Th2 cells differentiation, and IL-10 secretion); (III) *Streptococcus* promotes monocytes to produce Th1 and Th17 cells polarization factors; (IV) *Prevotella* stimulated Th17 cells by DC; (v) *Akkermansia* reduce the number of CTLs and Macs.

With the subdivision of studies on different gut microbiota, it has been found that even bacteria from the same phylum have different compositions and physiological activities. Therefore, each kind of bacteria has a unique physiological function. By acting on different immune cells, they regulate the function and gene expression of immune cells, thus playing a complex and diverse role in immunity. Gut microbiota also interact with each other, and the interaction between microbiota is an important factor in determining the final metabolites of gut microbiota (78). Although it has been proved that gut microbiota can act on IMC, few studies explored the potential mechanism of gut microbiota on IMC, and there is no mainstream theory about the improvement of IMC by gut microbiota. We generally summarize the following:(1) change the diversity and composition of gut microbiota; (2) affect mucosal barrier function; (3) regulate mucosal and systemic immunity. Here are some common intestinal bacteria and their metabolites that may affect the molecular mechanism of IMC (**Figure 2**).

### Actinobacteria

Actinobacteria play an important role in the breakdown of organic materials such as cellulose and chitin, thus in organic material turnover and carbon cycling. Actinobacteria are considered a treasure trove of secondary metabolites, including the *Streptomyces*, *Actinomycetes*, and so on. One of the most widely known is *Bifidobacterium*.

#### Bifidobacterium

*Bifidobacterium* known as a probiotic is used to improve many types of colitis, including IBD. What is more, it can also enhance the efficacy of ICIs while attenuating the toxic effects. Sun et al. first explored the mechanism of *Bifidobacterium* improving IMC and found that *Bifidobacterium* treatment on IMC mice increased the relative abundance of *Lactobacillus rhamnosum* and promoted self-stimulating circulation of IL-10/IL-10Rα in the intestine, inhibited effector T cells by enhancing Tregs function and reduces damage to normal tissues (78). *Bifidobacterium* not only affects the function of immune cells but also may affect the energy utilization of immune cells. *Bifidobacterium* enhances mitochondrial function and activity while increasing mitochondrial volume and mitochondrial stress in Treg cells (78). *Bifidobacterium adolescentis* can also improve DSS-induced colitis by stimulating protective Treg, Th2 cells response, and reshaping gut microbiota (79).

At the same time treated with *Bifidobacterium*, compared with wild-type mice, IL-10 knockout mice and IL-22 antibody-treated mice lost more weight, had more leukocyte infiltration in the colon, had more severe damage to the intestinal structure, and also increased the levels of inflammatory cytokines in serum. This indicates that IL-22 and IL-10 play a crucial part in *Bifidobacterium* improvement of IMC.

### Bacteroidetes

*Bacteroidetes* are an important part of intestinal bacteria, including *Bacteroides*, *Prevotella*, etc. They participate in many important metabolic activities in the colon. *Bacteroidales* promote the presence of IEL in the colon and then produce IL-6 in myeloid differentiation primary response 88(MyD88) dependent way to maintain intestinal epithelial barrier homeostasis (80).

#### Bacteroides

*Bacteroides thetaiotaomicron* (*B. thetaiotaomicron*) can alleviate colitis by regulating tryptophan metabolism and T cell differentiation in inflammatory intestinal tissues. *B. thetaiotaomicron* stimulates antigen-specific CD4 + T cells of *B. thetaiotaomicron* to differentiate into Treg cells and effector T cells. Consumption of *B. thetaiotaomicron*-specific Treg cells can lead to colitis (81). *B. thetaiotaomicron* promotes the preferential differentiation of anti-inflammatory Treg and Th2 cells while inhibiting the differentiation of pro-inflammatory Th1 and Th17 cells, thereby reducing colitis. At the molecular level, *B. thetaiotaomicron* is related to CpG methylation within the Foxp3 promoter, which can enhance Tregs function. *B. thetaiotaomicron* also increases the levels of indoleacetic acid and indole propionic acid to increase Aromatic hydrocarbon Receptor (AhR) activation, which is connected with changes in the expression profile of transcription factors in T cells (82). *Bacteroides fragilis*(*B. fragilis*) has the immunomodulatory effect of limiting inflammation by stimulating Treg cells differentiation and increasing IL-10 level (83). Colonization of *B. fragilis* can reduce the infiltration of inflammatory cells (macrophages and neutrophils) in the colon mucosa (84). Polysaccharide A(PSA) is a macromolecular carbohydrate, as well as an immunogenic capsule component of non-toxigenic B. fragilis. It is an immunomodulatory factor that can promote mucosal immune development and inhibit colitis. *B. fragilis* colonization alone was sufficient to redress the imbalance of Th1/Th2 cells in the spleen of germ-free mice. Besides, PSA alleviates colitis by inhibiting pro-inflammatory cytokines relevant to the Th17 cells lineage (85). PSA can also promote the transformation of CD4+T cells into Foxp3+Treg cells and the production of IL-10 (83). Toll-like receptor 2(TLR2) is a transmembrane receptor that recognizes the pathogen-associated molecular patterns such as PSA, initiating a signaling cascade to promote immune response. PSA induces and preferentially binds to TLR2 on plasmacytoid DC (pDC), and then pDC specifically stimulates CD4+ T cells to secrete IL-10. Therefore, PSA can affect the inflammatory state by regulating innate and adaptive immunity (86). In addition to *B. fragilis*, *Parabacteroides distasonis* can also stimulate DC and then induce CD4 + T cells to differentiate into Treg cells, playing a protective role in the acute colitic mouse model (87).

Interestingly, not all *Bacteroides* have a positive effect on IMC, and some *Bacteroides* seem to be involved in the pathogenesis of IMC. For example, Enterotoxigenic *B. fragilis* is a strain of B. fragilis that secretes a 20-kDa heat-labile zinc-dependent metalloprotease toxin termed the B. fragilis toxin. It is closely related to IBD and colorectal cancer, and can rapidly induce IL-17-dependent colitis and tumorigenesis (88). Oral administration of *Bacteroides ovatus* and *Bacteroides xylanisolvens* induces the expression of pro-inflammatory factors CXCL9 and IFN-γ in the mouse model of lung cancer (89).

#### Prevotella

*Prevotella* induced the production and accumulation of Th17 cells in the colon and increased Th17 cells related cytokines in serum. The interaction between *Prevotella* and TLR2 on DC mediates the production of Th17-polarizing cytokines (IL-6 and IL-1β), indicating the potential pathogenic role of *Prevotella* in the gut (90).

### Firmicutes

*Firmicutes* are the largest bacterial group in the intestinal tract, including *Bacillus*, *Enterococcus*, *Lactobacillus*, *Streptococcus*, *Clostridium*, etc. They are the main bacterial group that produces short-chain fatty acids. Multiple studies have shown that the enrichment of *Firmicutes* is connected with IMC. Therefore, except for *Lactobacillus*, many bacteria from *Firmicutes* will promote the occurrence and development of IMC. The depletion of Treg cells in mice is accompanied by an increase in the relative abundance of *Firmicutes* and the occurrence of intestinal inflammation in mice, indicating that the effects of Treg cells and microbes are mutual (91). The preclinical study conducted by Chaput et al. showed that IMC may be related to the increased proportion of ICOS+CD4+T cells and ICOS+Treg cells induced by *Firmicutes (* 55).

#### Lactobacillus

*Lactobacillus* is a kind of probiotic in firmicutes commonly used in fermented dairy products. Oral administration of *Lacticaseibacillus paracasei* significantly changes the microbial community and increases microbial diversity, helps to maintain intestinal homeostasis, and provides a complete intestinal barrier against IMC (92). *Lactobacillus rhamnosus* can reduce the severity of inflammation in the IMC mouse model by regulating Treg cells (93). Oral administration of *Lactobacillus rhamnosus* can activate the innate immunity of the host to enhance the immune response, regulate the ratio of Treg to Th1 population and the expression ratio of IL-10 and TNF-α to inhibit the host’s systemic inflammation (94). T-cells’ response can be indirectly manipulated by regulating intestinal DC. *Lactobacillus casei Shirota* can regulate DC in inflammatory intestinal tissue, which is manifested in the decrease of TLR2/4 expression, restoration of the ability to imprint homing molecules on T cells, and the stimulation of T cells to produce IL-22 (95). *Lactobacillus acidophilus* and *Clostridium butyricum* can reduce Th1-type colitis and Th2-type colitis, their combination can reduce neutrophil infiltration and improve colon ulcers (96). *Lactobacillus reuteri* mainly inhibits the occurrence and progression of IMC by reducing the number of ILC3 (44). *Lactobacillus fermentum* shows beneficial effects on colitic mice, which decreases levels of Th1, Th2 and Th17 cells related cytokines, and increases CD4+Foxp3+Treg cells in the colon and mesenteric lymph nodes (MLN). In addition, the administration of *Lactobacillus fermentum* reshapes and increases the diversity of gut microbiota, and increases the abundance of beneficial bacteria such as *Lactobacillus* and *Akkermansia (* 97). *Lactobacillus acidophilus* treatment can also alleviate the severity of DSS-induced colitis and inhibit pro-inflammatory cytokines in the colon, such as IL-1β、 IL-6、IL-17, and TNF-α. In addition, *Lactobacillus acidophilus* directly induces the production of Treg cells and IL-10 (98).

#### Streptococcus

*Streptococcus* spp. is known to be enriched in patients with irAEs (99). *Streptococcus salivarius* and *Streptococcus agalactiae* can effectively activate monocytes to secrete higher levels of IL-6, IL-12 and TNF, which are Th1 and Th17 cells skewing cytokines (100).

#### Clostridium

FMT can restore the balance of gut microbiota and alleviate colitis, especially up-regulate the abundance of *Lactobacillus* and down-regulate the abundance of *Clostridium* and *Turicibacter*. FMT can also selectively down-regulate the proportion of CD4+ and CD8+ T cells in the colon to maintain intestinal homeostasis. In addition, *Clostridium* is associated with inflammation-related genes CCL8 and IDO1 (101).

#### Other bacteria

The mixture of *Fusobacterium*, *Phascolarctobacterium*, *Eubacterium*, and *Ruminococcus* of *Firmicutes* showed obvious IFN-γ+ CD8 +T cell induction ability (102). MAIT cells are activated during bacterial infection and exert antibacterial effects by producing IFN-γ, TNF-α, and IL-17 (103). The increase of MAIT cells in IMC patients proves the connection between microbiome and IMC. The antibacterial activity of MAIT cells may lead to the destruction of the intestinal barrier and the impairment of immunoregulation in IMC patients. SCFAs producing bacteria are positively correlated with the number of MAIT cells, such as *Acidaminococcaceae* of *Firmicutes* and *Alistipes shahii* and Alistipes inops of *Bacteroidetes (* 104).

### Verrucomicrobia

Verrucomicrobia is a newly classified group of bacteria that includes only a handful of recognized species. As a representative of verrucomicrobia found in feces, *Akkermansia muciniphila* makes up only about 3% of the intestine, but it plays multiple roles. *Akkermansia muciniphila* can improve colitis by reducing infiltrating macrophages and CD8 + CTLs in the colon and MLN (105).

### Gut microflora metabolites

**Figure 3:**
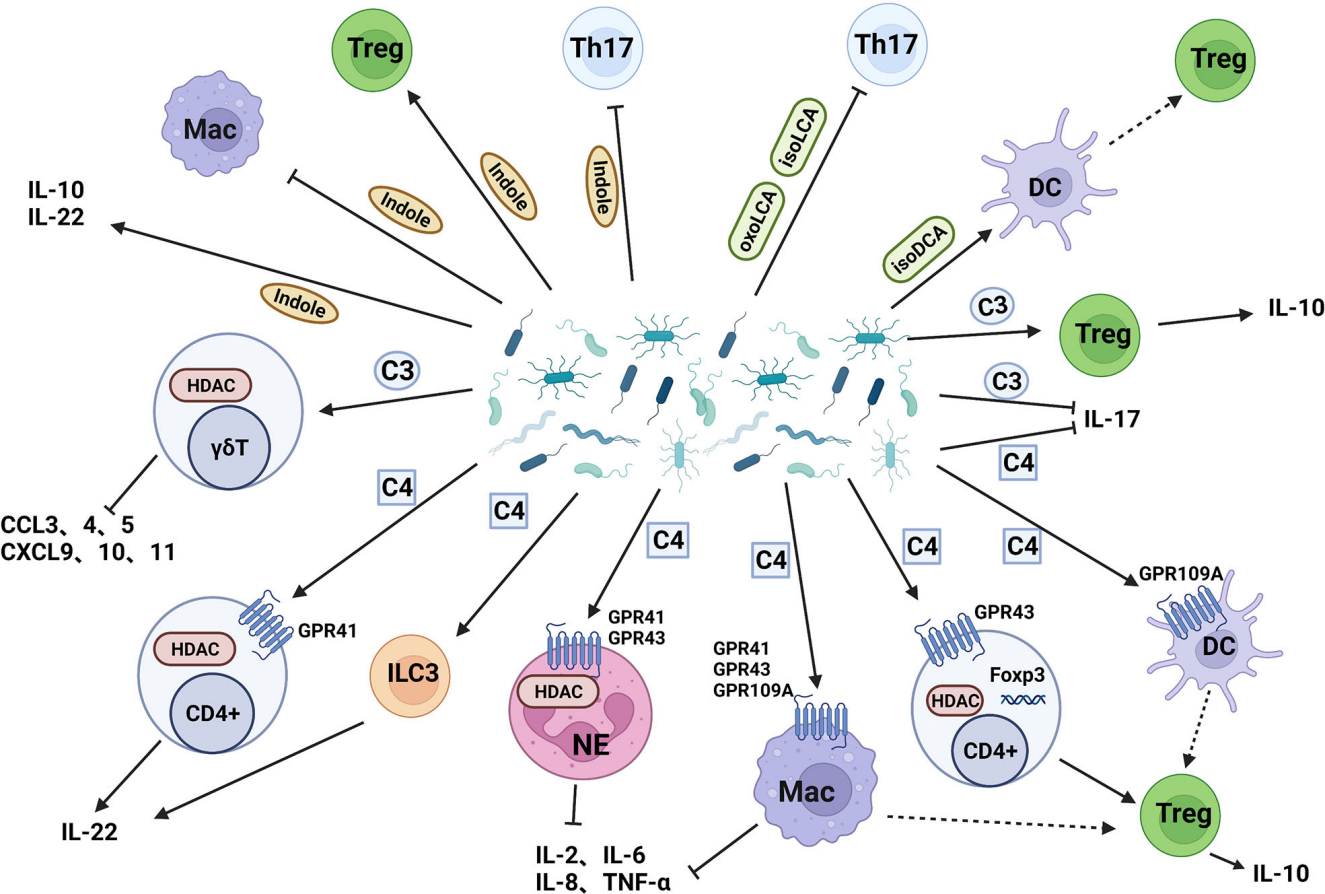
Intestinal bacterial metabolites act on IMC. (I) Butyrate acts on dendritic cells, neutrophils, and macrophages through GPR to induce Treg cells differentiation, reduce the release of proinflammatory cytokines, and induce Treg cells differentiation by inhibiting HDAC; (II)Propionate reduces the release of pro-inflammatory chemokines and promote the secretion of IL-10 by Treg cells; (III) Secondary bile acids inhibit Th17 cells and induce Treg cells production through dendritic cells; (IV) Indole inhibit Th17 cells and macrophages, induce Treg cells production, and improve intestinal cell junction and barrier function. C3: Propionate, C4: Butyrate.

The microbiota carries out complex and active metabolic activities in the human intestine, which not only provides energy and nutrition for growth but also produces a large number of metabolites such as short-chain fatty acids, secondary bile acids and indole derivatives into the human body. Short-chain fatty acids (SCFAs) are organic acids with 2-5 carbon atoms, which are mainly produced by gut microflora through fermentation and metabolism of oligosaccharides, polysaccharides, titanium, proteins, and glycoproteins (106). Bile acids (BAs) are cholesterol-derived molecules that participate in nutritional and energy-related physiological processes. Most of the BAs in the body are transported back to the liver through the enterohepatic circulation, but a small part of them are reabsorbed in the ileum and further transformed by bacteria in the colon, resulting in secondary BAs (107). About 4-6% of tryptophan moves along the gastrointestinal tract and is metabolized to indole and its derivatives by gut microflora (108). These metabolites affect intestinal homeostasis and inflammation by affecting immune cells and inflammatory factors (**Figure 3**).

#### Short-chain fatty acids

SCFAs mainly include acetic acid, propionic acid, isobutyric acid, butyrate, isovaleric acid, and valeric acid. Acetic acid is the main SCFA in the colon, and most *Enterococcus* can produce it; Propionic acid can be produced by *Bacteroides* and *Clostridium*; Butyrate mainly comes from *Clostridium* and *Eubacterium (* 106). SCFAs have an immunosuppressive effect on both innate and adaptive immunity. G-coupled protein receptor 41 (GPR41), GPR43, and GPR109A are specific SCFAs receptors in humans. SCFAs reduce the production of TNF-α、IL-2, IL-6, and IL-8 by inhibiting histone deacetylase(HDAC) and activating GPR41 and GPR43 on neutrophils and macrophages. SCFAs can also inhibit the recruitment of macrophages and neutrophils, indicating their potential anti-inflammatory effect *in vivo (* 109). Butyrate can regulate the function of intestinal macrophages. After treatment with butyrate, the proinflammatory mediators NO, IL-6 and IL-12 are down-regulated, maintaining the tolerance of macrophages to gut microbiota (110). Butyrate can also induce macrophages and DCs through GPR109A to regulate Treg cell amplification and increase the expression of IL-10 (111). Butyrate enhanced histone H3 acetylation in the promoter, and conserved non-coding sequence regions of the Foxp3 locus, induced the differentiation of Treg cells in the colon, and also improved the colitis caused by CD4 (+) T cells in mice (112). Similarly, some studies also found that butyrate increased the levels of Treg cells, IL-10 and IL-12 in peripheral blood of mice, decreased the levels of IL-17 in plasma and colon mucosa and thus improved colitis in mice (113). SCFAs promote the production of IL-22 by CD4 + T cells and ILC3 through inhibition of HDAC and GPR41 mediation (114). *Ffar2* is the gene encoding GPR43. The binding of GPR43 and SCFAs on immune cells can mediate the resolution of inflammatory responses (115). Studies have confirmed that SCFAs relies on the expression of Ffar2 to prevent and improve colitis in mouse (109). In addition to Treg cells, SCFAs can also act on other cells to affect intestinal homeostasis. SCFAs promote the secretion of IL-10 by Th1 cells through GPR43 mediation, thereby weakening the pathogenic ability of gut microbiota antigen-specific Th1 cells to induce intestinal inflammation (116).

IL-17A producing γδ T cells are tissue resident cells involved in tissue defense and intestinal inflammation regulation. Microbiota-derived propionate inhibits IL-17 production by intestinal γδ T cells in HDAC-dependent manner and has been successfully verified in IBD patients (117). In colitic mice fed with propionate, the number of Foxp3+Treg cells and the level of IL-10 in the colon lamina propria of increased, the weight of mice increased, the disease score decreased, and the levels of colon proinflammatory cytokines decreased (109). Butyrate and propionate regulate gene expression and lymphocyte recruitment in DC since SCFAs strongly reduce the release of several pro-inflammatory chemokines (118).

#### Secondary bile acids

Endogenous bile acids in the intestinal cavity can promote the renewal and regeneration of intestinal stem cells in response to injury (119). *Firmicutes*, *Actinomycetes*, and *Bacteroides* can convert secondary bile acids into 3-oxolitholic acid(3-oxoLCA) and isolithocholic acid (isoLCA), and then inhibit the differentiation of Th17 cells. The contents of 3-oxoLCA and isoLCA in IBD patients are also greatly reduced, indicating that this may be one of the mechanisms leading to intestinal inflammation (120). Secondary bile acid 3β-Hydrohydroxycholic (isoDCA) can increase the induction of Foxp3+Treg cells by reducing the immunostimulatory properties of DC. The microbiota communities containing engineered *Bacteroides* are designed by using synthetic biological methods, which can produce isoDCA and promote the generation of Treg cells *in vivo (* 121).

#### Indole and its derivatives

Indole and its derivatives bind to AhR, enhance barrier function and tight connection, maintain intestinal homeostasis, regulate intestinal immune tolerance and weaken inflammatory response, maintain the integrity of apical junction complex and related actin regulatory proteins to reduce intestinal permeability (122–124). Indole-3-acetic acid (I3A) and indole-3-methanol (I3C) produced by *Bacteroides ovatus* can promote immune cells to produce IL-22 and prevent microbial imbalance caused by colitis, thus have a beneficial impact on colitis (125). I3C can also increase butyrate production, inhibit Th17 cells and induce Tregs production to alleviate colitis (126). Indole reduces pro-inflammatory signals (IL-8 and NF-κB) and promotes the expression of anti-inflammatory ingredients including IL-10 (127). Both tryptamine and I3A reduce the production of proinflammatory cytokines in colon macrophages (128).

Intestinal adaptive immunity plays a crucial part in maintaining immune tolerance and the integrity of the intestinal barrier, and the innate immune system can regulate the adaptive immune response. Gut bacteria can not only directly affect the adaptive immune system, but also act on the innate immune system and thus affect adaptive immunity. Due to the variety of gut microbiota, each bacterium has different effects on IMC. Gut microbiota metabolites are indispensable physiological substances in the human body. With the deepening of its research, it is found that these metabolites also play a key role in inhibiting intestinal inflammation and maintaining intestinal homeostasis.

## Clinical application

Recently, a number of large-scale clinical trials for the application of gut microbiota and related products in IMC have been established to research the potential biomarkers in the microbiome and their safety and effectiveness in IMC treatment (**Table 2**). These different microbial components can be used to develop clinical biomarkers to divide patients into susceptible and non-susceptible groups to IMC before ICIs treatment, to balance the potential risks and benefits of immunotherapy for patients. It can also be used to alleviate and treat IMC, especially in hormone-insensitive cases.

**Table 2:** Clinical trials of intestinal microbiological intervention for IMC patients.

Clinical Trial number	Patient population	Intervention	ICI type	Goal	Outcomes
NCT04163289	Renal Cell Carcinoma	FMT	Ipilimumab Nivolumab	The safety of FMT combination treatment and reduce the occurrence of immune-related toxicities.	Occurrence of IMC, changes in patient microbiota, success rate of FMT.
NCT03819296	Any Cancer Type with IMC	FMT	Any	The role of the gut microbiome and FMT on immunotherapy-induced gastrointestinal complications.	Difference in stool microbiome pattern incidence of adverse events of FMT.
NCT04038619	Malignant Genitourinary System Neoplasm with IMC	FMT	Any	FMT works in treating IMC in patients with genitourinary cancer.	Safety (adverse effects) and clinical response (remission of colitis/diarrhea). Recurrent after FMT.
NCT03643289	Advanced Melanoma		Any	The effects of gut microbiome diversity on responses and side effects of immunotherapy in advanced melanoma patients.	Gut microbiota diversity and peripheral blood mononuclear cells immunophenotyping about responses to treatment and side effects in patients with stage 3 or stage 4 melanoma receiving immunotherapy.
NCT02600143	Melanoma		Ipilimumab	Identify biomarkers and genetic predisposition for the development of IMC.	Identify a genetic profile associated with IMC. Identify predictive (serum or fecal) biomarkers for IMC. The role of the gut microbiome in the development of IMC.
NCT04107311	Solid Tumor		Any	Evaluating the role of the intestinal microbiome and autoimmune panels as a predictor for irAEs.	Feasibility of evaluating intestinal microbiome composition and autoimmune panels in patients treated with Immuno-oncology combinations.
NCT04552418	Solid Tumor	Resistant starch (prebiotic)	Ipilimumab Nivolumab	Safety and feasibility of administering a dietary supplement to patients undergoing cancer treatment with dual ICIs for solid cancer.	Safety (adverse events) and feasibility(adherence). Incidence and severity of IMC, change in luminal microbiota composition and metabolite.

ORR, Objective response rate; QoL, Quality of life.

Antibiotics can change the composition and content of gut microbiota, eradicate a variety of pathogenic bacteria including *Helicobacter pylori*, and then prevent the occurrence and development of tumors. However, some studies have confirmed that antibiotics can reduce the efficacy of tumor immunotherapy, and the cumulative incidence rate of IMC is significantly higher in antibiotic-exposed patients. Therefore, antibiotics are considered a double-edged sword in tumor treatment (129). Antibiotic treatment for anaerobes will lead to the occurrence and progress of IMC, and the application of antibiotics after ICIs treatment has a higher incidence rate of IMC than that before ICIs treatment (130). Component analysis of the intestinal microbiome of melanoma mice showed that vancomycin aggravated the symptoms of IMC by killing *Lactobacillus*. Oral administration of *Lactobacillus reuteri* can inhibit the occurrence and progression of colitis, thereby improving the weight loss and inflammatory state caused by ICIs treatment (44). The use of antibiotics still needs further discussion, but antibiotics should certainly be used cautiously during immunotherapy.

FMT uses the feces of healthy donors from another individual or the same individual to balance or restore the intestinal microbial composition through oral gavage or direct delivery to the gastrointestinal tract. It is a safe and low incidence of adverse events of procedure, which holds great promise in the treatment of recurrent *Clostridium difficile* infection and ulcerative colitis (131). The potential of IMC insensitive to corticosteroids by changing the composition of gut microbiota has been reported. An IMC patient whose symptoms were not relieved after corticosteroids and infliximab treatment completely disappeared and never relapsed after FMT (132). Wang et al. reported two patients with glucocorticoid-resistant IMC whose symptoms were completely relieved after FMT treatment (133). In the future, if the efficacy of FMT is proved to be more beneficial than the current first-line treatment, FMT may be used as the first-line treatment of IMC. Nevertheless, because FMT is an invasive procedure and has not been used as a routine method for clinical treatment, investigational new drug applications should be submitted before FMT. So FMT used as the treatment of IMC has a long way to go. Researches are still needed to determine microbiota kinds, the frequency of FMT as well as the risks associated with FMT and other protocols and specifications (134). Currently, many clinical trials on the application of FMT are underway to investigate its safety and efficacy in the treatment of IMC(NCT04163289, NCT03819296, NCT04038619, see **Table 2** for details).

Prebiotics are various fibers that can be digested by microorganisms. Bacteria can metabolize prebiotics to produce secondary metabolites such as SCFAs. In prebiotic supplemented mice, the genus relative abundance of *Ruminococcus* and *Bifidobacteria* increased, which was related to significantly reducing tumorigenesis, increasing the concentration of SCFAs in cecum and feces, and reducing the expression of inflammatory markers (135). Feeding 2-Fucosyllactose to mice with colitis can decrease IL-1β and IL-6 expression, increase TGF-β and occludin expression and propionate concentration, accompanied by the expansion of *Ruminococcus*, and the most important thing is to effectively alleviate inflammation (136).Clinical trials on the application of prebiotics have been established to investigate its safety and feasibility in the treatment of IMC(NCT04552418, see **Table 2** for details).

Engineered microbiomes are new microorganisms that be engineered using modern bioengineering techniques such as genome editing. They have a variety of advantages, including site-specific drug delivery, sustainable release and convenient control, and a large number of studies have fully demonstrated their broad prospects in the treatment of metabolic diseases and cancer. Researchers constructed an engineered butyrate-producing strain, BsS-RS06551, using B. subtilis SCK6 as cellular chassis, could produce high levels of butyrate and increase the abundance of beneficial bacteria, such as *Bifidobacterium*, *Lactobacillus*, and *Akkermansia (* 137). Researchers engineered Escherichia coli Nissle 1917 (ECN) to express IL-10, produce curly fiber matrix, ketone body (R)-3-hydroxybutyrate (3HB), and Schistosoma immunomodulatory protein Sj16 and other substances. These substances can protect the intestinal mucosa by promoting the growth of probiotics, promoting intestinal epithelial integrity and repair, and downregulating inflammatory response-related cells or cytokines, so as to improve DSS-induced colitis (138–141).

## Conclusion

IMC is the most common irAEs, and its incidence rate varies with different types of ICIs. How to prevent and treat IMC is a difficult problem in clinical practice. In this review, we first introduce the onset cycle and clinical characteristics of IMC. Next, we discuss the pathogenesis of IMC, especially the abnormalities of the innate and adaptive immune systems. Then we summarize the latest progress in animal research and human clinical trials and demonstrate that the diversity and composition change of gut microbiota is an important factor of IMC. In addition, we focus on how some common gut bacteria affect IMC. Gut microbiota not only affects local immunity but also affects the systemic immune system. Each kind of bacteria has unique physiological activities, which can trigger anti-inflammatory or pro-inflammatory pathways by affecting the function and metabolism of different immune cells. Gut microbiota metabolites are also an important part of balancing intestinal homeostasis and inflammation. Finally, we summarize the clinical trials on the intervention of intestinal bacteria and related products in IMC patients, to explore the possibility of using gut microbiota as a biomarker of tumor immunotherapy toxicity. As well as exploring the effectiveness and safety of using FMT, prebiotics, probiotics, metabolites, and engineered microbiomes to avoid immunotherapy intestinal toxicity.

irAEs are caused by the excessive immune response attacking normal tissues, which usually means excessive activation of the anti-tumor immune response. Many studies have confirmed that anti-tumour response is higher in patients with IMC and other irAEs (142). Therefore, the methods used for the treatment of IMC may reduce the response to ICIs at the same time, which is not desirable. Many bacteria in the gut microbiota, such as *Bifidobacterium*, *Lactobacillus*, and *Bacteroides fragilis*, play an active role in enhancing the antitumor efficacy of ICIs and improving IMC. If the targeted microbiota modulation is performed before or during the treatment of ICIs, the antitumor effect of ICIs can be maximized and IMC can be minimized. However, there is still a lack of representative clinical trials to promote the transformation of research results into clinical practice. In the future, more detailed research is needed to determine the timing, duration, target bacteria, and specific methods of microbiota regulation. With the deepening study of gut microbiota, it may be possible to directly or indirectly regulate gut microbiota and its metabolites in the future, to achieve targeted regulation of immunotherapy, enhance therapeutic efficacy and reduce adverse reactions including IMC. This will enable more patients to benefit from immunotherapy in the long term and prolong the survival of tumor patients.

## Author contributions

XH was mainly responsible for writing conception, literature searching, and drafting of the manuscript. DZ provided professional revision for the article. DL participated in the collection of literature and revision of the article. JC participated in the conception and revision of the article. All authors contributed to the article and approved the submitted version.

## Funding

This work was supported by the Chinese Society of Clinical Oncology (CSCO) Research Foundation (Nos. Y-JS2019-034)and “1+X ”program for Clinical Competency enhancement–Clinical Research Incubation Project, The Second Hospital of Dalian Medical University(2022LCYJYB01)

## Acknowledgments

The authors are grateful to the directors (Prof. Jun Chen) and team members of the Department of Oncology, The Second Hospital of Dalian Medical University for the guidance and wonderful mentoring that led to the writing of this paper.

## Conflict of interest

The authors declare that the research was conducted in the absence of any commercial or financial relationships that could be construed as a potential conflict of interest.

## Publisher’s note

All claims expressed in this article are solely those of the authors and do not necessarily represent those of their affiliated organizations, or those of the publisher, the editors and the reviewers. Any product that may be evaluated in this article, or claim that may be made by its manufacturer, is not guaranteed or endorsed by the publisher.

## Abbreviations

ICIs, Immune Checkpoint Inhibitors; irAEs, Immune-Related Adverse Events; IMC, Immune Checkpoint Inhibitor-Mediated Colitis; CTLA-4, Cytotoxic T Lymphocyte Antigen-4; PD-1/PD-L1, Programmed Death-1/Programmed Death Ligand-1; FMT, Fecal microbiota transplantation; APCs, Antigen-presenting cells; Treg, Regulatory T; TCR, T-cell receptor; IBD, Inflammatory Bowel Disease; ILC3, Group 3 innate immune cells; IL, interleukin; MAIT, mucosa-associated invariant T; DSS, Dextran Sodium Sulfate; CTL, Cytotoxic T lymphocytes; Trm, Tissue resident memory T cells; CXCL, CXC motif chemokine ligand; IEC, Intestinal epithelial cells; Intraepithelial lymphocytes; GM-CSF, Granulocyte-macrophage colony-stimulating factor; CRP, C-reactive protein; sCTLA-4, Soluble CTLA-4; HLA, Human Leukocyte Antigen; CICB, Combined immune checkpoint blocking; MyD88, Myeloid differentiation primary response 88; PSA, Polysaccharide A; TLR2, Toll-like receptor 2; pDC, Plasmacytoid DC; MLN, Mesenteric lymph nodes; IDO, indoleamine*2,3*-dioxygenase; CCL, CC motif chemokine ligand; SCFAs, Short-chain fatty acids; Bas, Bile acids; GPR, G-coupled protein receptor; HDAC, Histone deacetylase; Th, T helper cell; Mac, macrophage; NE, neutrophil; DC, dendritic cell; MNC, monocyte; TNF, tumor necrosis factor; IFN, interferon.
